# Recreations

**Published:** 1858-05

**Authors:** 


### RECREATIONS.

ONE of the most useful articles we have lately written is that on Throat-Ail, in the April number. We have been deluged with affirmatory comments; and in reference to one of the topics introduced, a clergyman writes: "Two years ago, I started on a tour over Europe and the East, finished with Egypt, Arabia Petræa, Palestine, and Syria. I was gone over fifteen months; and felt well and happy all the while, when not sea-sick. Several weeks after my return, I took a severe cold, and had to work in spite of it. Was advised to recreate for a month, did so; but have not felt so strong as before, and take cold easily. Perhaps the change from a roving, bustling life was too sudden in my case; I saw the danger, and took great precaution, as I thought. My experience illustrates your remarks in the last two Journals. When I got through Syria, I was twenty pounds heavier than I had ever been before; then I took a steamer at *Beyroot* for home—pined, puked, and groaned, for nearly a month, over waves, and through storms, amid filth, and cabins crammed with foul air. When I reached New York, I had less flesh than when I started. Men are great fools to run to the ends of the earth to get health, which they cannot even bring along home; and if they should be so fortunate as to get it on thus far, they seldom have sense enough to keep it six months."

It may be useful to our medical subscribers to state, that this is not the first patient we have had who has returned from Palestine and Syria to be so grievously afflicted on returning to this country as to be incapacitated from the discharge of pastoral duty—there being either an ugly aguishness or such a bilious condition of the system as to prostrate the health entirely. In each of these cases, a persistent antibilious treatment—the vocal affection being pretty much left to take care of itself—has not failed certainly to be followed by an ability to fill pastoral duty. More cases of Throat-Ail are founded in a torpid liver, or an imperfect digestion, than one physician in a million has any conception of. In a week's treatment, on these principles, this gentleman feels as if he were going to get well.

